# Lack of executive functions deficits among adult ad hd individuals from a Brazilian clinical sample

**DOI:** 10.1590/S1980-57642009DN30100007

**Published:** 2009

**Authors:** Eloisa Saboya, Gabriel Coutinho, Daniel Segenreich, Vanessa Ayrão, Paulo Mattos

**Affiliations:** 1 Psicóloga, Doutora em Saúde Mental, Instituto de Psiquiatria da Universidade Federal do Rio de Janeiro, Rio de Janeiro, RJ, Brazil.; 2 Psicólogo, Mestre em Saúde Mental, Instituto de Psiquiatria da Universidade Federal do Rio de Janeiro e Centro de Neuropsicologia Aplicada do Rio de Janeiro, Rio de Janeiro, RJ, Brazil.; 3 Psiquiatra, Mestre em Psiquiatria, Instituto de Psiquiatria da Universidade Federal do Rio de Janeiro, Rio de Janeiro, RJ, Brazil.; 4 Psiquiatra, Pesquisadora do GEDA, Instituto de Psiquiatria da Universidade Federal do Rio de Janeiro, Rio de Janeiro, RJ, Brazil.; 5 Psiquiatra, Doutor em Psiquiatria, Professor Associado - Instituto de Psiquiatria da Universidade Federal do Rio de Janeiro, Rio de Janeiro, RJ, Brazil.

**Keywords:** Attention Deficit Hyperactivity Disorder, attention, executive functions, neuropsychological tests, adults

## Abstract

Executive function deficits have been previously documented in individuals with Attention Deficit Hyperactivity Disorder (ADHD).

**Objective:**

The current study aimed to compare measures of executive functions among a clinical sample of adults with ADHD and normal control subjects, matched for age, gender and education.

**Methods:**

Twenty-three self-referred adults diagnosed with ADHD according to DSM-IV criteria, and twenty-five control subjects were assessed using a neuropsychological battery which included the Wisconsin Card Sorting Test, Tower of Hanoi, Digit Span, Trail Making Test (A and B), Stroop Test and Raven’s Progressive Matrices.

**Results:**

The ADHD group did not differ significantly from the control subjects on any of the measures assessed.

**Conclusion:**

Measures of executive functions using this test battery were unable to discriminate between adults with ADHD and control subjects in this clinical sample.

## Abstract (pt)

Déficits de funções executivas foram previamente documentados em portadores de Transtorno do Déficit de Atenção e Hiperatividade (TDAH).

**Objetivo:**

O presente estudo objetivou comparar medidas de funções executivas entre adultos portadores de TDAH e controles normais, pareados por idade, sexo e escolaridade.

**Métodos:**

Vinte e três adultos auto-referidos diagnosticados como portadores de TDAH de acordo com os critérios do DSM-IV e vinte e cinco controles foram avaliados com bateria de testes neuropsicológicos, que incluía: Teste de Seleção de Cartas de Wisconsin, Torre de Hanoi, Amplitude de Dígitos , Teste das Trilhas (A e B), Teste de Stroop e Matrizes Progressivas de Raven.

**Resultados:**

O desempenho dos portadores de TDAH não diferiu de forma significativa dos controles em nenhum dos testes utilizados.

**Conclusão:**

A bateria de testes de funções executivas utilizada no presente estudo não foi capaz de discriminar adultos portadores de TDAH de controles desta amostra clínica.

Attention Deficit Hyperactivity Disorder (ADHD) has been conceived as a disorder in the development of executive functions (EFs) since the publication of Barkley’s hybrid model.^1^ Briefly, EFs account for a sophisticated system of diverse skills, which enable the individual to perform voluntary, independent, autonomous, self-organized and target-oriented actions.^2^ Some dysexecutive symptoms may also be related to comorbid conditions in ADHD; for example, impulsivity and inattention may be linked to Eating Disorders, which were first documented as highly comorbid in ADHD by our group.^3^

A meta-analysis study comparing neuropsychological executive and non-executive functioning between adults with ADHD and normal controls found that neuropsychological difficulties in the former group might be present in both executive and non-executive domains.^4^ Another meta-analysis conducted by Willcutt et al.^5^ concluded that although EF deficits were often present in ADHD subjects, these deficits were neither necessary nor sufficient to predict the presence of the disorder. Recently, Biederman et al.^6^ showed that self-reported questionnaires developed to evaluate EF deficits had better ecological validity than neuropsychological EF tests.

Studies investigating neuropsychological aspects of ADHD in adults are scarce in Brazil, where most research has sought to evaluate neuropsychological aspects of ADHD in children and adolescents. However, some groups have been attempting to evaluate ADHD adults with new tasks adapted for use in Brazil.^7^

The purpose of our study was to compare measures of EFs among a clinical sample of adults with ADHD versus normal control subjects, matched for age, gender and education. Based on the literature review, we hypothesized that ADHD subjects would perform worse on the executive measures in comparison to controls.

## Methods

Eighty-nine self-referred adults aged 18 to 59 years enrolled for treatment at the Attention-Deficit Study Group (GEDA) at the Institute of Psychiatry of the Federal University of Rio de Janeiro and underwent a comprehensive clinical assessment that included:

A) a modified childhood module for ADHD from the Kiddie SADS-E adapted for adults, in Portuguese;^8^
B) a psychiatric evaluation by a board-certified adult psychiatrist, including medical history and
C) comorbid axis I disorders, assessed using the Mini International Neuropsychiatric Interview (MINI).^9^

Past ADHD symptoms were investigated using the patients’ own recollections of a chronic course of ADHD symptoms (according to DSM-IV criteria).

The control group was recruited from a non-clinical population and comprised individuals who were invited to take part as volunteers. Some were undergraduates and others were volunteers from the local community. Each participant was included after being screened for any alcohol and/or drug abuse/dependence or any obvious symptoms of ADHD or depression.

All individuals were submitted to the following tests: Digit Span, both forward and backward as a working memory (WM) measure; Trail Making Tests A and B^10^ to evaluate visual-motor dexterity and conceptual flexibility; the Stroop Test^11^ as a control inhibition measure; Wisconsin Card Sorting Test (WCST)^12^ to test hypotheses and conceptual flexibility; Tower of Hanoi^13^ to evaluate planning and solving capacity, and Raven Progressive Matrices^14^ as a measure of fluid intelligence. ADHD individuals only started pharmacological treatment after completing the tests. None of them were in use of psychostimulants or other pharmacological treatment for ADHD at the time of the neuropsychological evaluation.

The study was approved by the institutional review board, and all subjects signed a written informed consent term before inclusion in the study.

### Statistical analysis

Comparison between neuropsychological test scores for the ADHD group and control group was performed using nonparametric analysis and the Mann-Whitney U test. Pearson’s Chi-Square test was employed for the gender variable, and the Mann-Whitney U test was applied for age and number of years in formal schooling.

## Results

Of 89 self-referred adults interviewed consecutively, 53 met the DSM-IV criteria for ADHD. Out of these 53 positively diagnosed adults, those with comorbid major depression and/or any drug or alcohol abuse or dependency, and/or who declared they were regular users of psychoactive substances were excluded (30 patients).

Forty-eight adults took part in the neuropsychological assessment. Twenty-three comprised the ADHD group whereas 25 were normal controls. The ADHD group comprised 8 male (34.8%) and 15 female subjects (65.2%). Their ages ranged from 18 to 59 years, with a mean age of 31.87 years and a standard deviation of 10.55 years. Formal schooling ranged from 5 to 21 years, with a mean of 14.13 years and a standard deviation of 3.15 years. The control group comprised 9 men (36%) and 16 women (64%) aged between 22 and 48 years (mean: 33.24 years and standard deviation: 8.21 years). Formal schooling ranged between 7 and 21 years, with a mean of 13.36 years and a standard deviation of 3.64 years (Table 1).

**Table 1:** Comparison between ADHD and control groups for gender, age and years of education.

Group	Total	Gender				Age			Years of education	
		Male	Female	*p* value *		Mean rank	*p* value *		Mean rank	*p* value *
ADHD	23	8	15	**0.930**		22.57	**0.357**		26.65	**0.300**
Control	25	9	16			26.28			22.52	
Total	48	17	31							

* Gender significance was calculated using Pearson's Chi-Square test; Number of years in formal schooling and age significance were calculated using the Mann-Whitney test.

No statistically significant differences were found between the groups (*p*>0.05) for gender, age, or formal schooling. Results are shown in Table 1.

We found no differences between ADHD and control groups in neuropsychological tests results compared using nonparametric analysis (U-test). No statistically significant difference was found for any of the neuropsychological variables (*p*>0.05). Table 2 shows the values of ranks and statistics from the U-test.

**Table 2:** Ranks and Mann-Whitney Test.

Variables	Group	Mean rank	*p* value*
RPM - total score	ADHD Control	27.17 22.04	0.203
Digits forward	ADHD Control	24.48 24.52	0.992
Digits backwards	ADHD Control	24.33 24.66	0.933
Digits total score	ADHD Control	24.24 24.74	0.901
Span forward	ADHD Control	23.61 25.32	0.658
Span backwards	ADHD Control	24.09 24.88	0.840
Trail A - time	ADHD Control	26.02 23.10	0.470
Trail B - time	ADHD Control	23.80 25.14	0.741
TOH - movements	ADHD Control	22.08 23.74	0.671
Stroop	ADHD Control	22.30 24.60	0.556
WCST perseverative errors	ADHD Control	20.33 26.16	0.133
WCST categories	ADHD Control	26.67 20.84	0.124
WCST failure to maintain set	ADHD Control	20.76 25.80	0.124

RPM, Raven Progressive Matrices; TOH, Tower of Hanoi; WCST, Wisconsin Card Sorting Test.

* Significance was calculated using the Mann-Whitney test (U-test).

## Discussion

The lack of any statistically significant difference between the ADHD and the control group for the three socio-demographic variables – gender, age and years in formal schooling – as well as general fluid intelligence, allows the conclusion to be drawn that none of these variables systematically influenced the results.

Further, no significant difference was observed between the two groups for any of the neuropsychological variables, which might suggest:

A) the test battery employed was not sensitive in differentiating patients from controls or
B) the magnitude of the difference between patients and controls was small and may only be demonstrated by a much larger sample or
C) our sample was biased in toward a group of individuals without significative functional impairment.

Some authors have suggested that the lack of differences found in studies that have used classic tests of EFs should be expected since such measures were not developed specifically to evaluate persons with ADHD.^15^ McGough and Barkley^16^ suggested that studies using neuropsychological tests of EFs have not yet demonstrated sufficient positive or negative predictive power to allow their recommendation for clinical use in children or adults. Also, a meta-analysis study^5^ has shown that EF deficits are not necessary nor sufficient to predict all cases of ADHD, which might suggest that negative results are to be expected in some situations. Recently, Biederman et al.^17^ demonstrated that impaired performances on EFs tasks in ADHD individuals were more strongly linked to lower IQ levels than to impairment in everyday activities that depend on EFs, which were shown to be better predicted by self-reported questionnaires devised to assess EF deficits.

It is likely that the participants of the present study represent a high-performance ADHD group in view of their length of schooling (around 14 years’ schooling, which in our setting implies university education) together with the absence of comorbidity with depression and the use of substances. Also, Biederman et al.^6^ have shown that neuropsychological measures aimed to evaluate EFs were more prone to record deficits associated to low IQ, whereas self-report questionnaires that evaluate EF deficits were better suited for functional impairments due to dysexecutive syndrome.

Interpretations of the present results must take into consideration the small sample size and the consequent low statistical power. Also, a clinical self-referred sample does not necessarily mirror aspects that might be found in non-clinical samples. The higher prevalence of female patients must also be taken into account when interpreting the results. Moreover, we did not exclude some important axis-I comorbid conditions that could have influenced test performance, such as Generalized Anxiety Disorder or Eating Disorders. Also, our test battery did not include some tests demonstrated to be more sensitive and/or ecological, such as Conners’ Continuous Performance Test, the Iowa Gambling Test or even self-reported questionnaires for EFs. Future studies using ecological tests and self-reported questionnaires for EF deficits should be performed in order to broaden the current investigation and bring new contributions to the theoretical knowledge and clinical management of executive dysfunctions in ADHD.

Our findings suggest that the neuropsychological battery employed was unable to discriminate between adults with ADHD and controls. These results might be related to low sensitivity of the neuropsychological tests or a small magnitude of difference detectable only in much larger samples. An alternative explanation is that our sample was comprised of ADHD persons with a high performance level, which may have minimized differences between groups. This explanation is supported by other studies that have previously suggested that ADHD subjects with above-average IQs may not differ significantly from normal comparison subjects in terms of EFs.^18^
